# Real-time in vivo rectal wall dosimetry using MO*Skin* detectors during linac based stereotactic radiotherapy with rectal displacement

**DOI:** 10.1186/s13014-017-0781-4

**Published:** 2017-02-27

**Authors:** Kimberley Legge, Peter B. Greer, Daryl J. O’Connor, Lee Wilton, Matthew Richardson, Perry Hunter, Alex Wilfert, Jarad Martin, Anatoly Rosenfeld, Dean Cutajar

**Affiliations:** 1 University of Newcastle, University Drive, Callaghan, 2308 NSW Australia; 2 Calvary Mater Newcastle, Cnr Edith and Platt Streets, Waratah, 2298 NSW Australia; 3 University of Wollongong, Wollongong, 2522 NSW Australia

**Keywords:** Prostate cancer, MOSFET detector, In vivo dosimetry, Stereotactic radiation therapy

## Abstract

**Background:**

MOSFET dosimetry is a method that has been used to measure in-vivo doses during brachytherapy treatments and during linac based radiotherapy treatment. Rectal displacement devices (RDDs) allow for safe dose escalation for prostate cancer treatment. This study used dual MOSkin detectors to assess real-time in vivo rectal wall dose in patients with an RDD in place during a high dose prostate stereotactic body radiation therapy (SBRT) boost trial.

**Methods:**

The PROMETHEUS study commenced in 2014 and provides a prostate SBRT boost dose with a RDD in place. Twelve patients received two boost fractions of 9.5–10 Gy each delivered to the prostate with a dual arc volumetric modulated arc therapy (VMAT) technique. Two MOSkins in a face-to-face arrangement (dual MOSkin) were used to decrease angular dependence. A dual MOSkin was attached to the anterior surface of the Rectafix and read out at 1 Hz during each treatment. The planned dose at each measurement point was exported from the planning system and compared with the measured dose. The root mean square error normalised to the total planned dose was calculated for each measurement point and treatment arc for the entire course of treatment.

**Results:**

The average difference between the measured and planned doses over the whole course of treatment for all arcs measured was 9.7% with a standard deviation of 3.6%. The cumulative MO*Skin* reading was lower than the total planned dose for 64% of the arcs measured. The average difference between the final measured and final planned doses for all arcs measured was 3.4% of the final planned dose, with a standard deviation of 10.3%.

**Conclusions:**

MO*Skin* detectors were an effective tool for measuring dose delivered to the anterior rectal wall in real time during prostate SBRT boost treatments for the purpose of both ensuring the rectal doses remain within acceptable limits during the treatment and for the verification of final rectal doses.

## Background

Prostate cancer shows a radiation response consistent with a low α/β ratio, indicating that hypofractionated treatment schedules may increase the effectiveness of treatment [1, 2]. Hypofractionation includes the delivery of higher dose fractions to boost the total dose prior to or following conventionally fractionated treatment. Delivery of a boost dose using high dose rate (HDR) brachytherapy in combination with conventionally fractionated external beam radiation therapy (EBRT) has been shown to improve tumour control rates when compared with conventionally fractionated EBRT alone [3–5]. EBRT is far more accessible than brachytherapy in Australia, and low rates of rectal and urinary toxicity along with good biological response rates have been observed in patients who received a boost dose delivered using an SBRT technique [6–8].

**Fig. 1:**
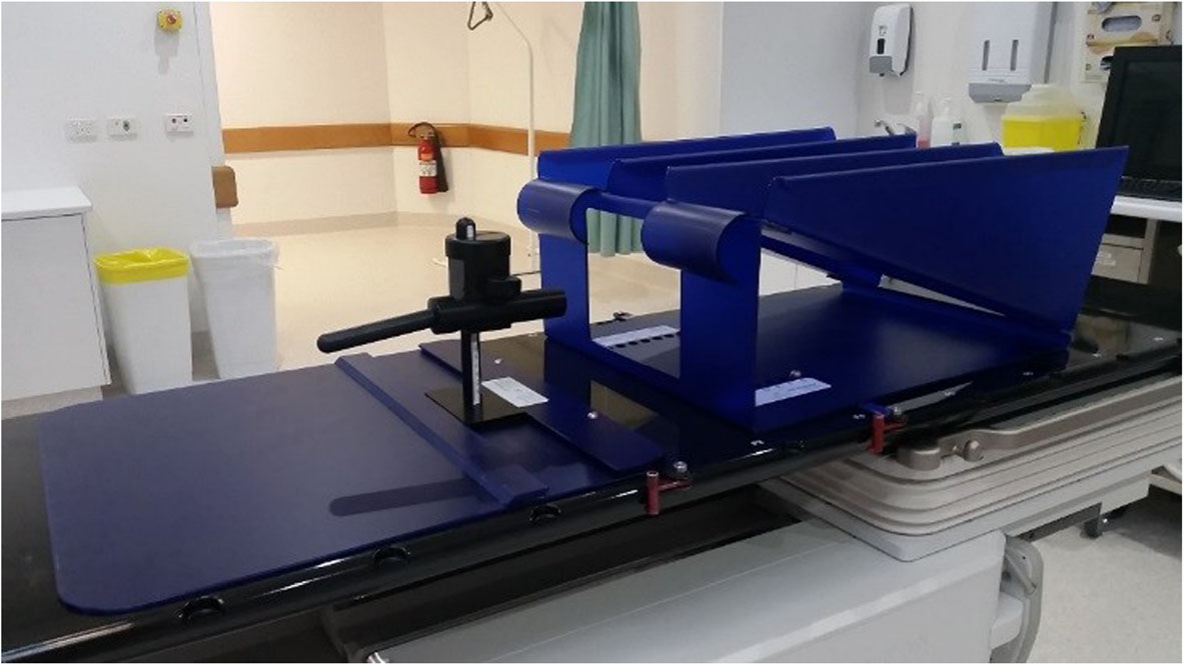
The Rectafix system

Rectal complications can arise as a result of radiation therapy for prostate cancer, and delivery of higher doses through hypofractionation increases the risk of damage to surrounding healthy tissues [9]. The risk and severity of rectal toxicities has been correlated with the volume of rectal wall exposed to high doses of radiation [10]. Use of dose volume constraints in planning and daily imaging to enable reduced margins are the most effective ways to reduce rectal dose, but rectal sparing devices such as injected spacer materials and the Rectafix rectal retractor have also been used to allow for safe dose escalation [11]. The Rectafix system (Fig. 1) uses a rod which is inserted into the patient’s rectum. The rod is then moved posteriorly on the vertical column and locked in place to manually move the rectum away from the prostate. The vertical column is marked to allow reproducible retraction. The Rectafix has been found to provide an average increase in separation of 0.5 cm and to assist in immobilising the rectal wall by preventing changes in filling by gas or faeces [12].

Changes in bladder or rectal volume during treatment can affect the dose coverage of the prostate and increase the dose to healthy tissues. One way to monitor the effect of any motion is real-time in-vivo dosimetric monitoring during treatment. Previous studies in this area have used plastic scintillation detectors and MOSFET based dosimeters in phantoms [13–16]. Plastic scintillation detectors attached to an endorectal balloon inserted in an anthropomorphic prostate phantom were used by Archambault et. al. to provide dose measurements every 150 ms during eight field intensity modulated radiation therapy treatments. In a field by field comparison, plastic scintillation detectors were found to agree with treatment planning system doses to within 0.5% [13]. MO*Skin* detectors are MOSFET based dosimeters. They have successfully been used to measure urethral dose inside a catheter placed within a gel phantom simulating a typical HDR brachytherapy treatment, with two MO*Skin* detectors used in a face to face arrangement (a dual MO*Skin*) found to be more accurate than a single MO*Skin* detector [14]. Dose to the anterior rectal wall in a phantom study of tomotherapy prostate boost dose while attached to a replica Rectafix rod has also been verified with MO*Skin*s, with all dual MO*Skin*s used in this study agreeing with treatment planning system dose to within ±5% when placed in a high dose gradient region [15]. MO*Skin*s have been integrated into a transrectal ultrasound probe of the kind used to guide interstitial needle insertion during HDR brachytherapy treatment. MO*Skin* measurements taken during three HDR brachytherapy treatment deliveries to a phantom resulted in an average discrepancy of −0.6 ± 2.6% between the measured and planned doses [16].

There have been very limited in-vivo patient dosimetry studies, however, in-vivo measurements have been performed during HDR brachytherapy treatments and EBRT treatments. Plastic scintillation detectors attached to an endorectal balloon were used by Wootton et al. to measure dose to the rectal wall over 142 prostate EBRT fractions for five patients. The average difference between measured doses and doses calculated on day of treatment CT scans was −0.4 ± 2.8% [17]. In a study of 12 patients by Carrara et. al., MO*Skin*s integrated into a transrectal ultrasound probe were shown to accurately measure dose delivered to the rectal wall during HDR brachytherapy treatments. The MO*Skin* measured doses were found to align more closely with delivered doses reconstructed after treatment than with planned doses in this case. The average absolute difference between MO*Skin* measured doses and reconstructed doses was 3.6  ±  1.9%, while the difference between MO*Skin* measured doses and planned doses was 6.7  ±  5.1%, with a total measurement uncertainty of 5.7%, demonstrating their potential for use in detecting treatment delivery errors [18]. However, assessment of rectal dose delivered during EBRT boost treatments using MO*Skin* detectors within patients has not been investigated to date, and previous studies have only compared measured doses with total planned doses for each field or treatment.

The aim of this work was to examine the feasibility of determining real-time in vivo rectal wall dose during prostate SBRT with a rectal retractor by monitoring during treatment with a dual MO*Skin* detector. Measured doses were compared with planned doses across the whole course of each treatment arc delivered to provide a measure of the accuracy of delivery, including treatment plan dose calculation and patient setup errors.

## Methods

1. Patient and treatment details
  The PROstate Multicentre External beam radioTHErapy Using Stereotactic boost (PROMETHEUS) study commenced in late 2014 and provides an EBRT boost dose consisting of two fractions to patients prior to a conventionally fractionated course of EBRT. A Rectafix rectal sparing device was in place during both boost fractions for the patients in this study. Twelve patients with intermediate to high risk prostate cancer (median age 72) consented to the ethics board approved study. EBRT boost treatments were planned using the Eclipse treatment planning system (version 11) and were delivered using either a Varian Clinac iX linear accelerator with a 6 MV photon beam at a maximum dose rate of 600 MU/min or using a Varian Truebeam linear accelerator with a 10 MV beam with a maximum dose rate of 600 MU/min or 10 MV flattening filter free photon beam with a maximum dose rate of 2400 MU/min. Six patients received a boost dose of 9.5 Gy per fraction and six patients received a boost dose of 10 Gy per fraction. All patients were treated with a Rectafix system in place. The Rectafix system is shown in Fig. 1. Patients received a pre-treatment cone beam computed tomography (CBCT) scan to check Rectafix positioning and bladder and rectal filling and were aligned using implanted gold fiducial markers. The prostate position in patients treated on the Truebeam was monitored using triggered two dimensional kilovoltage images taken every 3 s during treatment. Mid-treatment CBCT scans for repositioning were taken for all patients treated on the Clinac, and for patients treated on the Truebeam for whom the triggered imaging indicated prostate displacement had occurred. All patients received a post-treatment CBCT scan.
2. MO*Skin* Design
  MO*Skin* detectors, developed at the Centre for Medical Radiation Physics, University of Wollongong, are MOSFET based detectors designed to measure dose at the air-skin interface and have an active volume of 4.8 × 10^−6^ mm^3^ with a gate oxide thickness of 0.55 μm [19] and a water equivalent depth of 0.07 mm. This small volume makes them desirable for measurements in high dose gradient regions, [20] such as the anterior rectal wall in a prostate SBRT boost plan. MO*Skin* detectors provide temperature independent measurements between 15 and 40 °C [21] and have reproducible sensitivity for fraction sizes up to 10 Gy [20]. Two MO*Skin*s in a face-to-face arrangement (a dual MO*Skin*) have been found to reduce angular dependence resulting from the geometrical construction of the detector [22]. The dual MO*Skin* threshold voltage can be read out at 1 Hz and real-time readout software developed by the University of Wollongong can be used to convert the voltage to dose and display the cumulative dose delivered to the MO*Skin* as treatment progresses. MO*Skin* detectors are visible on cone beam CT (CBCT) scans. The MO*Skin* readout unit a dual MO*Skin* attached to the Rectafix rod appear in Fig. 2.

**Fig. 2:**
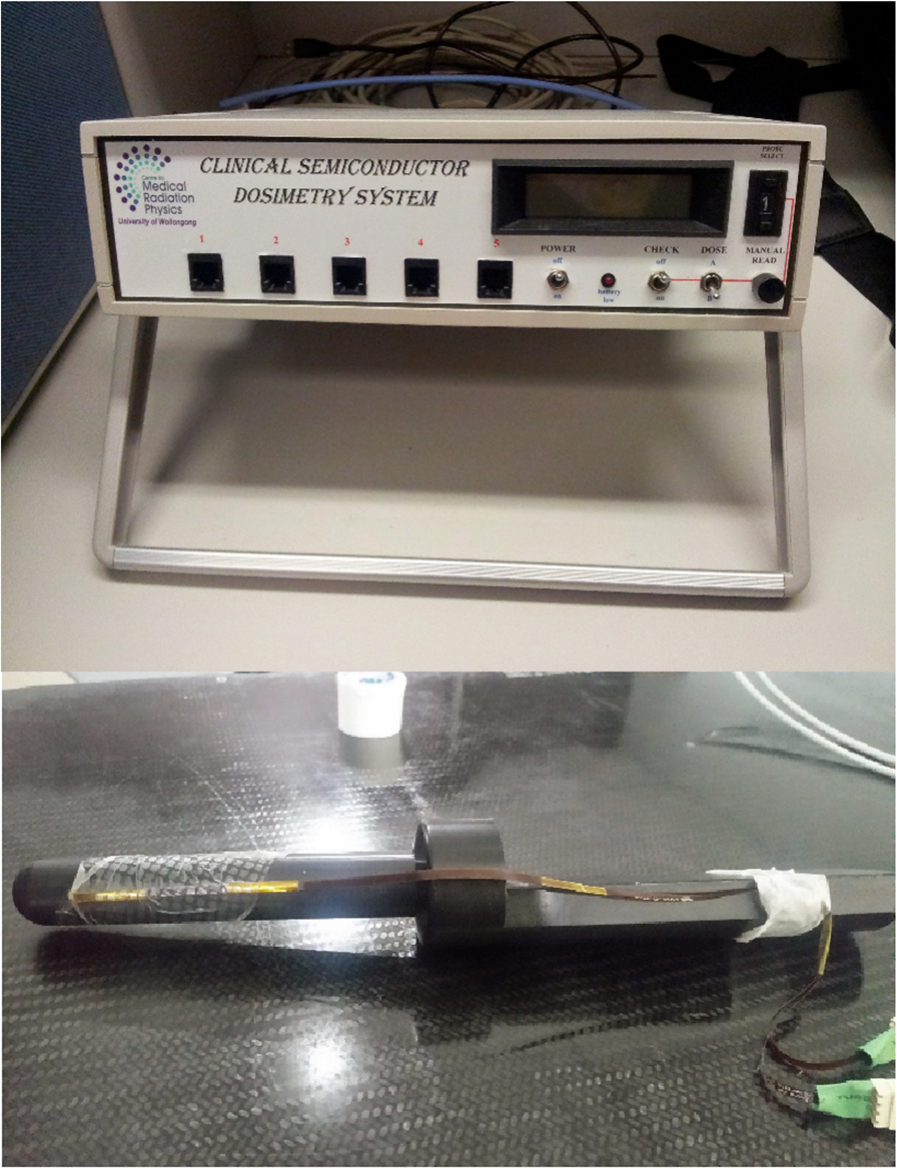
The Clinical Semiconductor Dosimetry System used to read out MO*Skin* detectors and a dual MO*Skin* detector attached to the Rectafix rod
3. Calibration and Testing
  Prior to treatment using the linear accelerator, dual MO*Skin* detectors were calibrated at the energy and dose rate that was to be used for patient treatment. Dual MO*Skin*s were aligned to isocentre using lasers and placed under 1.5 cm of solid water for 6 MV treatments, and under 2.5 cm of solid water for 10 MV treatments, with a 100 cm source to surface distance, so that the MO*Skin* would lie at the depth of dose maximum. A field size of 10 cm × 10 cm and collimator angle of 0° was used. Calibration was performed by delivering 20 cGy, as determined by nominal machine output, to the dual MOSkin arrangement in five separate irradiations. The dual MO*Skin* was then inverted, placing both detectors in inverse orientation so that the other detector was closest to the beam, and another five irradiations of 20 cGy were delivered.
  To determine a calibration factor for each dual MO*Skin*, the reading for each individual MO*Skin* detector at 30 s after beam off was recorded for each delivery, resulting in 20 total voltage values, five from each detector (×2 detectors) in the initial orientation and five from each detector in the inverse orientation. The 20 values obtained were then averaged. A calibration factor, CF (mV/cGy) was then obtained by dividing the average threshold voltage shift (ΔV_av_ mV) by the delivered dose (20 cGy) as shown in Eq. 1.
  1 $$ \boldsymbol{C}\boldsymbol{F}=\frac{\boldsymbol{\varDelta} {\boldsymbol{V}}_{\boldsymbol{av}}\  mV}{20\  cGy} $$
  Dual MO*Skin* detectors were tested for angular dependence by irradiation in the centre of a solid water cylinder 20 cm in diameter. The cylinder was raised 5 cm from the couch using solid water blocks for supports. The dual MO*Skin* was rotated relative to the beam and irradiated after every 30° rotation. MO*Skin* detectors were also tested for dose linearity from 10 to 200 cGy and dose rate dependence from 100 MU/min to 600 MU/min at 1.5 cm depth in solid water using a Varian Clinac iX linear accelerator.
4. Phantom Study
  As a final end-to-end test, a dual MO*Skin* was placed in a specially made insert in a CIRS 801-P Virtually Human Male Pelvis phantom (CIRS Inc., Norfolk, VA). A CT scan of the phantom with the insert in position was acquired and a 9.5 Gy dual arc VMAT plan was transferred to the CT scan and calculated. The treatment plan on the phantom with the specially made insert is shown in Fig. 3.

**Fig. 3:**
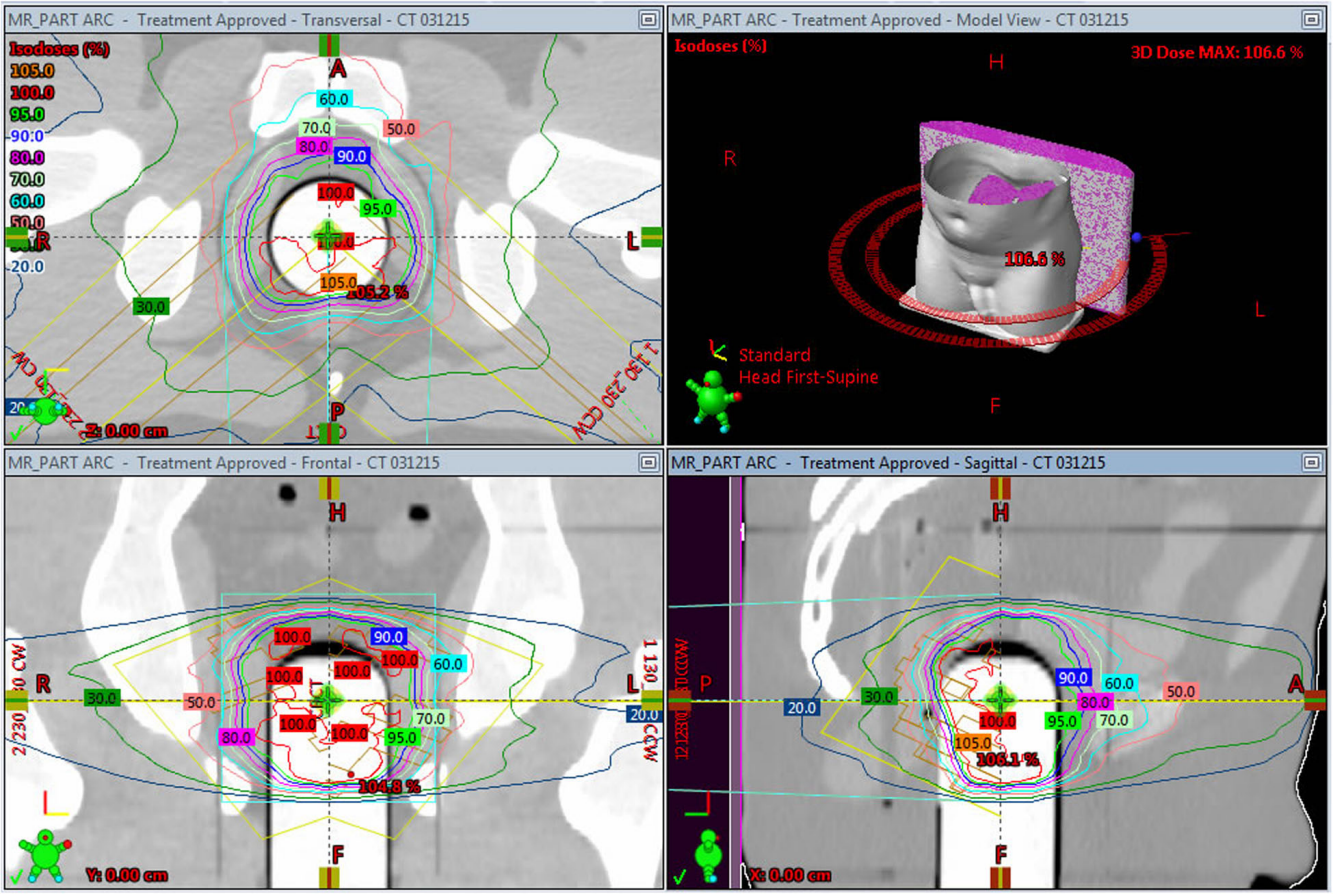
Dose plan on phantom with specially made insert for dual MOSkin detector
  The phantom was aligned with CBCT using a phantom insert with fiducial markers. This insert was then carefully removed and the insert with a dual MO*Skin* device was placed in the phantom. The plan was delivered using a Varian Clinac iX linear accelerator and the dual MO*Skin* was read out at 1 Hz during delivery. The same steps as outlined below in part six were used to compare the MO*Skin* measured dose with the planned dose extracted from the treatment planning system.
5. Treatment Delivery
  Prior to each treatment fraction, a dual MO*Skin* detector was attached to the anterior surface of the Rectafix rod using sterile adhesive wraps, as shown in Fig. 2. The entire rod was then covered with a condom to ensure that the risk of patient contact with the device was minimised. Each dual MO*Skin* was used for a single treatment fraction and then discarded. The location of the MO*Skin* was chosen by measuring within the Eclipse planning system, in the superior-inferior direction, the distance from the axial plane in which the collar of the rod appears to the axial plane in which the centre of the planning target volume (PTV) occurs so that the location was reproducible and as close to the centre of the PTV as possible.
  The dual MO*Skin* was attached to the clinical semiconductor dosimetry system, which was attached to a laptop computer. Real-time readout software called FETch (Centre for Medical Radiation Physics, University of Wollongong) was used on the laptop for readout. The Rectafix, with attached dual MO*Skin*, was inserted into the patient prior to alignment. Detectors were inside the patient for over 10 min prior to irradiation, giving the detectors time to equalise with patient temperature. Detectors were zeroed immediately prior to treatment delivery and voltages read out from both detectors in the dual MO*Skin* at 1 Hz during delivery of each two arc treatment. EPID images were acquired during treatment at 15.26 Hz using a research framegrabber computer. The gantry angle for each image was recorded by the imaging system so that the position of the gantry with time was known.
  Following delivery, MO*Skin* readouts were imported into a spreadsheet. The doses recorded by each of the individual detectors making up the dual detector were averaged and the result divided by the detector’s calibration factor.
6. Extraction of Planned Dose and Comparison
  Within the Eclipse treatment planning system, the location of the active area of the MOSFET in the CBCT acquired at the end of each fraction was marked. Each of these CBCTs was then manually registered to the patient’s planning CT. A verification point was then placed on the planning CT at the location of the MOSFET, yielding two verification points per patient, one for each prostate SBRT boost fraction. Figure 4 shows a post-treatment CBCT scan of a patient with the active area of the MO*Skin* marked.

**Fig. 4:**
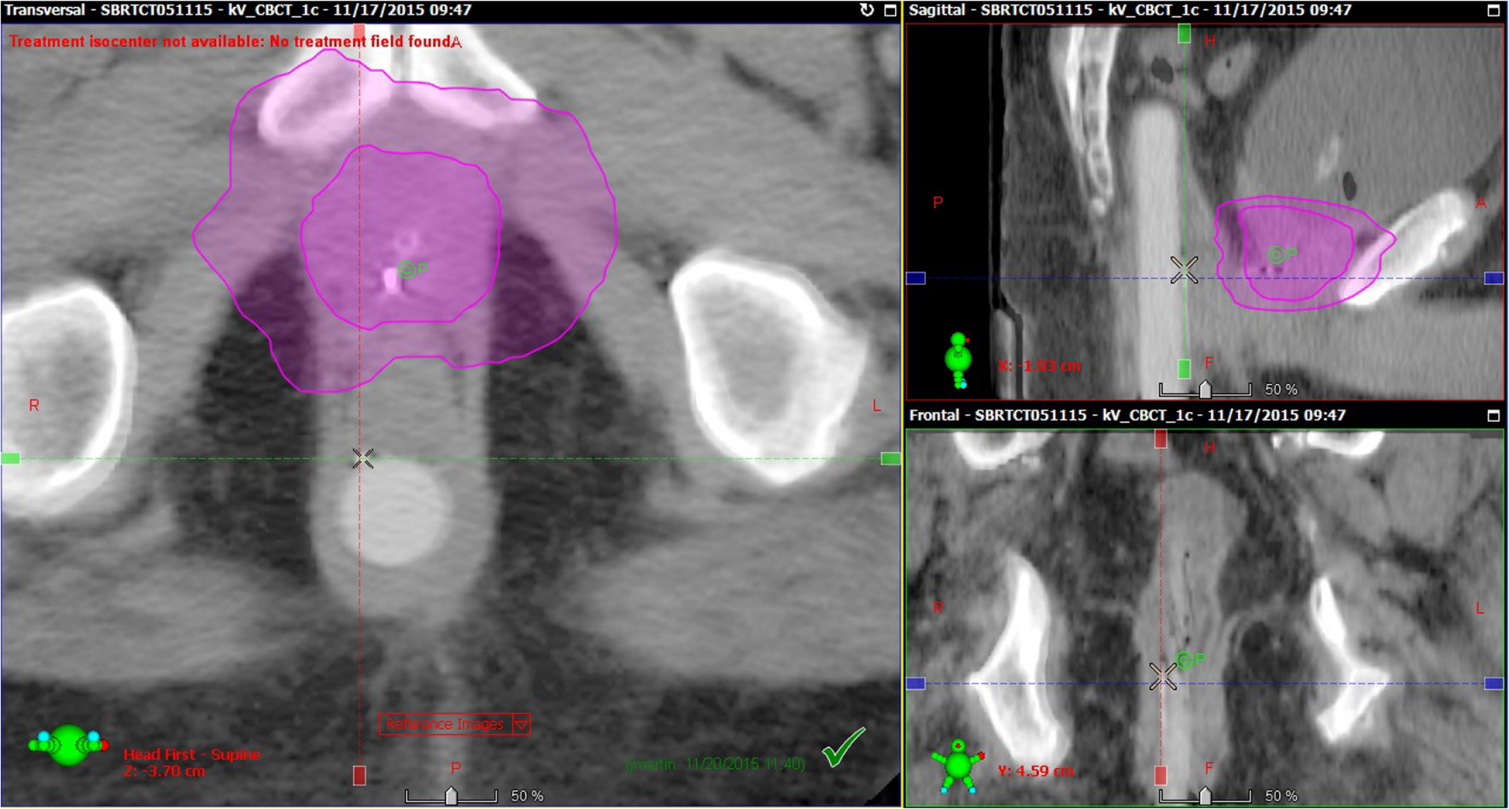
Marker at MO*Skin* location on CBCT scan taken following a patient treatment. The CTV and 50% isodose regions appear in *purple*
  For patients treated using a 6 MV or 10 MV beam with a flattening filter, a verification plan was created in the Eclipse treatment planning system with the treatment split into sub-arcs, each of 5.2°. The dose file for each sub-arc and the plan file were then exported from the system. For 10 MV flattening filter free beams, the version of the planning system available was unable to automatically split patient plans into sub-arcs. Five partial treatment plans were created for each of these patients, evenly splitting each arc by control points to obtain five cumulative dose points for each treatment. Following export of all dose and plan files, in-house software was used to extract the dose and gantry angle for each arc at the location of the MO*Skin* verification points. The position of the gantry with time was determined using spline interpolation from the gantry angles recorded in the header of the EPID images acquired at 15.26 Hz during treatment.
  Planned dose at the verification point and MO*Skin* measured dose were then plotted as a function of time over the whole course of each treatment fraction. The root mean square error (RMSE) was found for each treatment fraction to compare the MO*Skin* measured dose with the planning system dose across the whole treatment time. The RMSE value was normalised to the final planning system dose for each fraction to enable comparison between different patient plans.

## Results

1. Calibration and Testing
  The average calibration factor obtained for the dual MO*Skin*s used for phantom and patient treatments was 2.30 ± 0.07 mV/cGy. The maximum range of MO*Skin* responses for dose rates varying from 100 MU/min to 600 MU/min was ±2.6% for all dose rates measured. When varying the dose delivered, all MO*Skin* readings were within ±2.0%, indicating a linear response to dose.
  The results of the angular dependence testing appear in Fig. 5. The error bars are one standard deviation from the mean of two measurements taken at each angle. The MO*Skin* readings were normalised to the average MO*Skin* response for angles from 0 to 330°. All measurements were within ± 2.9% of the average MO*Skin* reading.

**Fig. 5:**
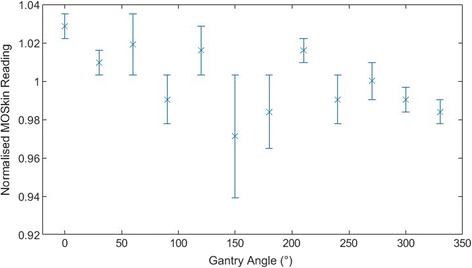
Angular dependence results for dual MOSkin detector
2. Phantom Study
  The results of the measurements performed in the anthropomorphic phantom appear in Table 1. The final dose difference was obtained by calculating the cumulative measured dose minus the final planned dose.

**Table 1:** Comparison of measured and planned doses for dual MO*Skin* measurements in an anthropomorphic phantom

Treatment Arc	Normalised RMSE (% of final planned dose)	Total dose difference (cGy)	Final dose difference (%)
Arc 1	3.3	1.1	2.4
Arc 2	2.1	−2.7	−6.4
  Figure 6 presents the dose measured by the dual MO*Skin* inserted into the anthropomorphic phantom and the planned dose at the MO*Skin* location.

**Fig. 6:**
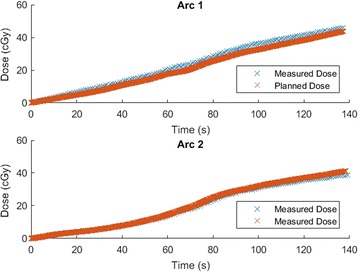
Plots of measured and planned dose obtained during delivery of a dual arc plan to a dual MO*Skin* in an anthropomorphic phantom
3. Patient Results
  Table 2 presents the normalised RMSE as a percentage of final planned dose for each patient treatment. RMSE comparison was performed so that the practicality of using MO*Skin* detectors to validate dose delivery in real time could be assessed. RMSE compares the measured and planned doses across the entire treatment delivery. The position of the MO*Skin* in fraction one was an average of 1.08 ± 0.66 cm from the position of the MO*Skin* during fraction 2.

**Table 2:** Normalised RMSE comparison of measured and planned doses for each treatment fraction

Patient	Normalised RMSE Fraction 1 (% of final planned dose)	Normalised RMSE Fraction 2 (% of final planned dose)
1	8.9	13.2
2	11.8	8.4
3	11.4	13.9
4	7.6	17.3
5	5.3	8.5
6	9.3	15.0
7	15.0	–
8	4.6	6.6
9	7.0	15.5
10	8.4	6.5
11	6.4	6.7
12	8.7	7.8
Average	9.7	
Standard deviation	3.6
  The average RMSE difference between the measured and planned system doses determined over all fractions measured in this study was 9.7% with a standard deviation of 3.6%. The cumulative MO*Skin* reading was lower than the total planned dose for 64% of the arcs measured. This is illustrated in Fig. 7, which shows the cumulative measured dose minus the total planned dose as a percentage of total planned dose for each patient arc. The average difference between the final measured and final planned doses for all arcs measured was −3.4% of the final planned dose, with a standard deviation of 10.3%. The average distance to agreement from the MO*Skin* location on the plan was 0.14 ± 0.11 cm across all patients, with a maximum distance to agreement of 0.47 cm.

**Fig. 7:**
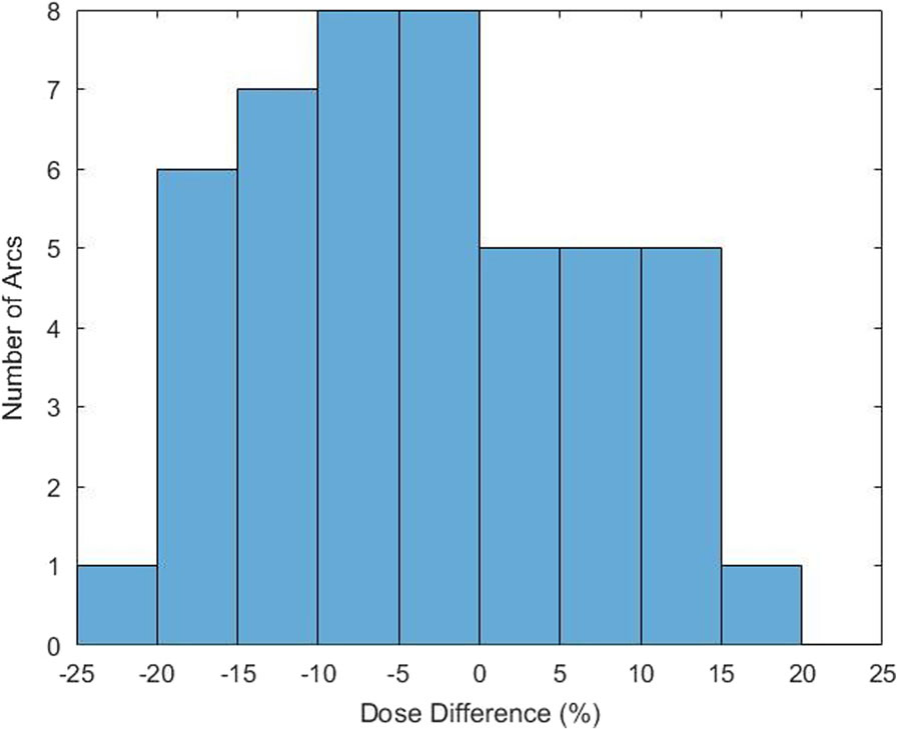
Comparison of total planned dose and cumulative measured dose per arc. The dose difference was calculated by determining the cumulative measured dose minus the total planned dose for each treatment arc
  Figure 8 shows the comparison of measured and planned dose for a patient treated using a 6 MV beam, so the plan was automatically split into sub-arcs of 5.2° in the Eclipse planning system.

**Fig. 8:**
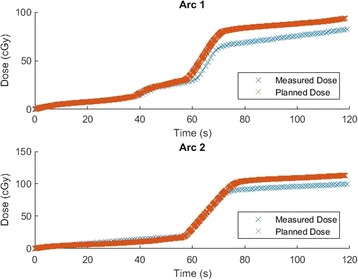
Comparison of planned and MO*Skin* measured dose to the anterior rectal wall as a function of time for each arc of a 9.5 Gy prostate SBRT boost treatment delivered with a 6X flattened beam. The planned dose has been exported in sub-arcs of 5.2°

## Discussion

MO*Skin* detectors attached to a Rectafix were used to measure anterior rectal wall dose in real time during prostate SBRT boost treatments. Measured doses were compared with planned doses extracted at the position of the MO*Skin* post treatment. Small modifications to the method used in this study could allow the delivered dose to be compared to the planned dose in real time during treatment, allowing for patient repositioning if the measured dose exceeds a preselected dose level during treatment.

The angular dependence measurements for the MO*Skin* performed in this study found a variation of ± 2.9% in MO*Skin* dose measurements taken every 30° of rotation. These results are in reasonable agreement with those measured in a previous study using dual MO*Skins* by Hardcastle et al., [20] who found a variation of ± 2.5% in readings performed in rotation increments of 30°. The discrepancy in these values may be due to differing geometry in the phantom setup or statistical fluctuations.

Uncertainty in the position of the MO*Skin* detector and verification point are major sources of discrepancy, as the detector is placed in a high dose gradient region during treatment. Across the rectal wall, the dose can decrease by 4.3% over a distance of 1 mm, as measured in the treatment planning system, meaning that a small discrepancy in the positioning of the verification point in the plan could mean a large difference in the dose exported from the planning system. While the MO*Skin* is visible on CBCT scans, precisely placing the verification point on the active area of the MOSkin is difficult, as the detector appears as a long strip with no delineation between the active area and the tail of the MO*Skin*, and the CBCT resolution makes it difficult to discern the exact location of the end of the detector. Patient shifts occurred between treatment arcs in nearly all cases measured, and patient anatomy in the area of the prostate is mobile, therefore, the verification point selected from the end of treatment CBCT does not indicate the location of the MO*Skin* detector throughout the entire course of treatment. The average difference between the MO*Skin* location on the pre-treatment CBCT and the post-treatment CBCT was 0.55 ± 0.47 cm which could result in a dose difference in that location of over 20%. The average distance to agreement of 0.14 ± 0.11 cm is well within this change in MO*Skin* location, indicating that the dose discrepancies likely result from the changing position of the MO*Skin* throughout the treatment due to patient motion and repositioning based on imaging. No fluctuations in detector signal stability were observed due to patient motion.

Previous studies using MO*Skin*s to measure dose in phantoms [14, 16] during brachytherapy reported better agreement between total measured and planned doses than the patient measurements reported here, as did a phantom study of rectal wall dose measurement during tomotherapy [15]. A phantom study using plastic scintillation detectors attached to endorectal balloons found agreement between measured and planned doses to within 0.5%, [13] and plastic scintillation detectors measured rectal wall dose in patients during EBRT with the mean difference between measured and planned doses for each patient ranging from −3.3 to 3.3% [17]. Measurements taken using MO*Skins* in patients during brachytherapy treatments found an average discrepancy between measured and planned doses of 6.3% with a standard deviation of 4.7% [18]. The results of the in-patient measurements reported here give a closer average agreement between measured and planned doses than the previous MO*Skin* study, 3.6%, with a larger standard deviation of 10.3%.

To adapt the method used here to allow for comparison of measured and planned dose in real time during treatment, Rectafix and MO*Skin* positioning would need to be consistent at simulation and for each fraction. At present, Rectafix positioning is guided by patient tolerance on the day of treatment. Preparation prior to treatment would require less than half an hour of work per patient to calibrate the MO*Skin* detectors and export the planned dose at the location of the MO*Skin* on the simulation CT. The planned dose with time could then be imported into the MO*Skin* readout software prior to treatment. The delivered dose could then be monitored in real time and adjustments made if the measured dose differed from the planned dose by a predetermined amount. Without these adjustments, the dose tolerance for any interruptions would need to be large, as small differences in the MO*Skin* position result in large dose differences.

## Conclusions

MO*Skin* detectors were used to measure dose to the anterior rectal wall in real time during prostate SBRT boost treatments. The average RMSE dose difference over the entire course of all arcs measured during this study was 9.7% with a standard deviation of 3.6%. Small modifications to the method presented here would allow MO*Skin*s to provide a dose alarm during SBRT treatments to avoid increased dose to the rectum in the case of delivery errors.

## Abbreviations

CBCT: Cone beam computed tomography
EBRT: External beam radiation therapy
HDR: High dose rate
RDD: Rectal displacement device
RMSE: Root mean square error
SBRT: Stereotactic body radiation therapy
VMAT: Volumetric modulated arc therapy
